# Medication-related hospital admissions and readmissions in older patients: an overview of literature

**DOI:** 10.1007/s11096-020-01040-1

**Published:** 2020-05-30

**Authors:** A. E. M. J. H. Linkens, V. Milosevic, P. H. M. van der Kuy, V. H. Damen-Hendriks, C. Mestres Gonzalvo, K. P. G. M. Hurkens

**Affiliations:** 1 Department of Internal Medicine, Maastricht University Medical Centre, PO Box 5800, 6202 AZ Maastricht, The Netherlands; 2 Department of Clinical Pharmacy, Pharmacology and Toxicology, Zuyderland Medical Centre, PO box 5500, 6130 MB Sittard, The Netherlands; 3 Department of Clinical Pharmacy, Erasmus Medical Centre, Postbus 2040, 3000 CA Rotterdam, The Netherlands; 4 Department of Internal Medicine, Zuyderland Medical Centre, PO box 5500, 6130 MB Sittard, Geleen, The Netherlands; 5 Department of Clinical Pharmacy, Maastricht University Medical Centre, PO Box 5800, 6202 AZ Maastricht, The Netherlands

**Keywords:** Admissions, Elderly, Medication, Polypharmacy, Readmissions

## Abstract

*Background* The number of medication related hospital admissions and readmissions are increasing over the years due to the ageing population. Medication related hospital admissions and readmissions lead to decreased quality of life and high healthcare costs. *Aim of the review *To assess what is currently known about medication related hospital admissions, medication related hospital readmissions, their risk factors, and possible interventions which reduce medication related hospital readmissions. *Method *We searched PubMed for articles about the topic medication related hospital admissions and readmissions. Overall 54 studies were selected for the overview of literature. *Results *Between the different selected studies there was much heterogeneity in definitions for medication related admission and readmissions, in study population and the way studies were performed. Multiple risk factors are found in the studies for example: polypharmacy, comorbidities, therapy non adherence, cognitive impairment, depending living situation, high risk medications and higher age. Different interventions are studied to reduce the number of medication related readmission, some of these interventions may reduce the readmissions like the participation of a pharmacist, education programmes and transition-of-care interventions and the use of digital assistance in the form of Clinical Decision Support Systems. However the methods and the results of these interventions show heterogeneity in the different researches. *Conclusion *There is much heterogeneity in incidence and definitions for both medication related hospital admissions and readmissions. Some risk factors are known for medication related admissions and readmissions such as polypharmacy, older age and additional diseases. Known interventions that could possibly lead to a decrease in medication related hospital readmissions are spare being the involvement of a pharmacist, education programs and transition-care interventions the most mentioned ones although controversial results have been reported. More research is needed to gather more information on this topic.

## Impact on practice

- Medication related admissions and medication related readmissions are common, however we still do not know enough to reduce them.
- Defining a common definition for medication related admissions and medication related readmission may ensure less heterogeneity in the future studies.

## Introduction

Thousands of medical interventions are performed each day in healthcare to improve the health status of our patients. The prescription of medication is an important intervention within the medical care for older patients [1, 2]. The rising incidence of multimorbidity and consequently polypharmacy adds to the complexity of managing older patients in particular [3]. Inadequate medication management and polypharmacy are important risk factors for adverse drug events and drug-drug interactions and frequently lead to hospital admissions and hospital readmissions and other undesirable consequences such as increased morbidity, decreased self-reliance and even death [4–7].

The number of acute and medication related hospital admissions is increasing over the years due to the ageing population [8]. In medication related hospital admissions two categories can be distinguished, namely primary admissions and readmissions. Less research is performed in the latter category. Both admissions and readmissions account for decreased quality of life and high healthcare costs [9, 10].

## Aim of the review

To give an overview on what is currently known about medication related hospital admissions, medication related hospital readmissions, their risk factors, and possible interventions which reduce medication related hospital readmissions.

## Methods

### Search strategy

We performed an overview of literature but not a systematic review. The single data source used was PubMed. We searched for articles with a set of MeSH terms and text words selected to cover articles on medication related admissions and medication related readmissions. The search was limited for articles published in English language. The search was performed in February 2017, with no limitations with regard to the publication date. We included articles that investigated the incidence of medication related admissions and medication related readmissions and their risk factors. We also included articles that investigated possible interventions which may reduce the rate of medication related readmissions. We selected studies that were performed in hospitals. We did not differentiate between hospital types for the performed studies. All study designs were allowed. The outcomes of the selected articles were dependant of the study. It was important that the outcome was related to the incidence of the medication related admissions and readmissions or their risk factors. Studies which investigated possible intervention to reduce the readmissions were also included.

We first selected articles based on the title. After the first selection two authors (AL and KH) independently assessed the articles for usability based on the abstract of the articles. Excluded were articles investigating an intervention or a treatment for a disease in which they had as a primary or secondary outcome the readmission rate. The quality of the different studies was not an exclusion criteria. When there was disagreement on in/exclusion of an article, a third reviewer was consulted and consensus was reached.

## Results

**Fig. 1:**
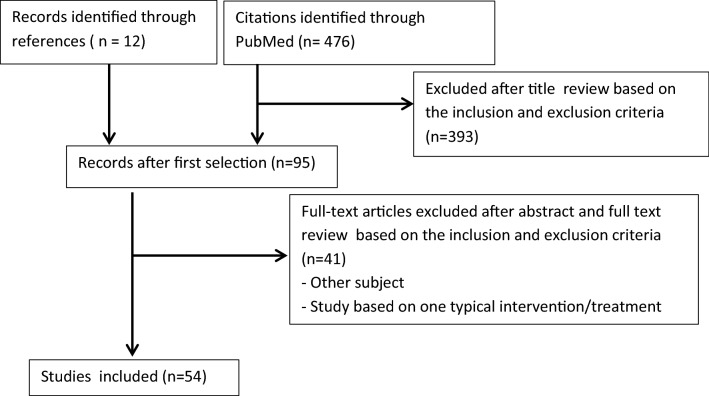
Flow diagram of the selection of studies for this literature overview

In total 476 records were retrieved with the PubMed search and we selected 12 records through references. Figure 1 shows the selection of the studies used for this literature overview. Overall 54 studies were assessed as relevant for the overview of this topic. In most of the excluded articles, the objective did not match our topic.

### Medication related hospital admissions

Medication related problems are a daily occurrence at the emergency department.

However, incidence rates on hospital admissions due to medication related problems differ because of the lack of a clear definition and the lack of identification which may underestimate the problem [11].

The most commonly used definition is an admission due to an adverse drug reaction (ADR). ADR is defined as: “a response to a drug that is noxious and unintended and occurs at doses normally used in man for the prophylaxis, diagnosis or therapy of disease, or for modification of physiological function” [12].

Another accepted definition of a medication related hospital admission is an admission due to an adverse drug event (ADE): “any untoward medical occurrence that may present during treatment with a pharmaceutical product but which does not necessarily have a causal relationship with this treatment” [13].

Finally, medication related hospital admissions are also defined as admissions due to drug related problems (DRP) [14, 15]. A DRP is defined as an event or circumstance that involves a patient’s drug treatment that actually, or potentially, interferes with the achievement of an optimal outcome [14–16].

Studies on the incidence of the association between hospital admissions and the presence of an ADR or ADE show great variety (0.5–18.9% and 5.6–19.3% respectively) [7, 10, 17–22].

A possible reason for the wide range of incidences is, as mentioned above, the variability in the used definition for medication related hospital admissions. Since ADE and DRP comprises a broader set of possible problems compared to ADR, incidence numbers could be higher. Also, patient inclusion criteria differ between studies. Some studies include all adults while other studies only include patients above 60 years old [10, 17, 19–22]. This influences the interpretation and comparability of results.

Furthermore, the type of patients differs between studies; where some studies include all unplanned admissions, other studies only include patients admitted for a specific ward [10, 17, 19, 21, 22].

Apart from the different inclusion and exclusion criteria, the study methods are also different. Different kind of designs and the selection procedure are used which may lead to a lack of identification of a medication related admission [17, 19, 21, 23].

In conclusion, there was much heterogeneity between studies in study population and the way studies were performed [22]. Although there was great variety in incidence, overall studies showed that medication related hospital admissions are a significant and possible preventable cause of unfavourable outcome and high healthcare costs.

### Medication related hospital readmissions

A hospital readmission is a second admission to the hospital within a certain period of time. In literature, different time periods are being used between the hospital discharge and readmission, ranging from 30 days to three years. However, a period of 30 days is most common [24–29]. Worldwide, readmissions are an important indicator for quality of healthcare and is therefore also part of the basic set of quality indicators of the Dutch Healthcare inspectorate (IGJ) [30].

As with medication related hospital admissions, different definitions of medication related readmissions are being used with regard to ADRs, ADEs and DRPs. A common definition assumes that medication-related hospital readmissions are readmissions due to problems around pharmacotherapy [31]. Different DRPs can occur in patients using medication, especially patients with polypharmacy. Examples for DRPs are problems with medication adherence, ADRs, inappropriate drug selection, drug use without indication, drug-drug interactions, additional therapy needed, lack of therapy monitoring, sub-therapeutic dosage and supra-therapeutic dosage. All problems in these categories can lead to a medication-related hospital readmission [31].

Another definition assumes medication related readmissions based on ADRs and ADEs [24, 32].

Because of the differences in definition, incidences of medication related readmissions vary greatly and range from 0.09% to 64.0% [27–29].

According to the definition you would expect that the rate for medication related readmissions based on ADRs is lower compared to ADE. The prevalence for the ADRs related hospital admission varies from 0.5 to 18.9% [17–22] and for de ADEs related hospital admission varies from 5.6% till 19.3% [7, 23].

Besides the variety in definitions used in the studies for medication related hospital readmissions, the time between discharge and readmission also differs between studies, ranging from 30 days to three years [24–29]. This makes interpretation of the results difficult. To conclude, studies with regard to hospital readmissions are also difficult to interpret due to differences in study design and definitions used.

### Risk factors for medication related admission and medication related readmissions

Several risk factors have been identified in medication related hospital admissions due to ADE’s. According to Leendertse et al. patients with impaired cognition, four or more comorbidities, dependent living situation, polypharmacy, impaired renal function and/or nonadherence to the medication regimen were found to be at greater risk of a hospital admission [7, 10]. These risk factors are consistent with other studies performed on this topic [23, 33]. The most common drugs associated with (potentially preventable) admissions were anticoagulants, antiplatelet drugs, vasodilators, psychotropic medications and diuretics [7, 23, 33]. Little is known about the risk factors of a medication related readmission for older patients but it is likely that there is great overlap with the risk factors for medication related admissions. Possible additional risk factors are a higher Charles comorbidity score and inadequate follow-up due to a missed appointment with the successive physician [24, 27, 28]. Increased age is also found as a risk factor of ADRs or ADPs related readmissions [25, 26].

Some specific medications are also associated with a higher incidence of medication related hospital readmissions. The most frequent medications which are associated with medication related hospital readmission are antiplatelet medications, diuretics, anti-coagulants and anti-hypertensive drugs [25, 29]. Zhang et al. found a greater risk for repeated ADRs for the drug categories hormones, primarily systemic agents (including antineoplastic, immunosuppresses and neoplastic antibiotics) and bacterial vaccines, resulting in a hospital readmission or an ADR during hospitalisation [24]. Alassaad et al. found in patients above 80 years old that drugs prescribed for peptic ulcer or gastroesophageal reflux disease and opioids are associated with an increased risk for readmission, the reason for the readmission was not mentioned [34]. Beside a higher risk for medication related readmissions due to specific medications, some studies also investigate the association between the complexity of the medication list and medication related hospital readmissions [35, 36]. The Medication Regimen Complexity Index (MRCI) is a frequently used score to predict the complexity of the medication regimen. It is based on 65 items and considers dosing frequency, dosage forms and other characteristics which may influence the complexity [37, 38]. A higher MRCI score reflects a more complex medication regime. Although a clear association was not established, most studies show a higher readmission rate when patients have higher MRCI [36, 38–40].

Olson et al. investigated if specific older patient populations are at risk for medication related hospital readmission [41]. They found that older men with adult children as caregiver seemed to have an increased risk for hospital readmissions [41]. Possible explanations are e.g. difficulties with regard to medication adherence due to parents who want to maintain their autonomy, or in case of siblings sharing the care for parents there could be confusion about the responsibilities. Another explanation is that medication problems could be related to informal caregiving itself. More research is needed to investigate the reason why males with adult children as caregivers show an increased risk for hospital readmissions.

Other risk factors which are associated with a higher readmission rate are low adherence, experiencing a fall in the last 12 months, weight loss and medical error due to discontinuity of care from inpatient to outpatient setting [42–45]. Table 1 shows the different published risk factors.

**Table 1:** Risk factors for medication related admissions and medication related readmissions

Risk factor	Studied in the following population	Found in the studies
High risk medication	Adult patients hospitalization at general medicine	Allaudeen et al. [44]
	50 years old and older and had one of a selection comorbidity	Schoonover et al. [39]
	Adult patients	Willson et al. [38]
	Adult patients with heart failure	Colavecchia et al. [36]
Polypharmacy	Patients 70 years old or older	Wimmer et al. [40]
	Patients hospitalized in a geriatric unit	Cabre et al. [33]
	Adult patients	McLachlan et al. [23]
Low or intermediate therapy adherence (combined)/non adherence	Patients above 65 years old or ten or more medications, heart failure, pharmacist consultation of duplications in medication list	Rosen et al. [42]
	Adult patients	Leendertse et al. [7]
Inappropriate medication	Patients hospitalized in a geriatric unit	Cabre et al. [33]
No pharmacy consult	Adult patients	Thomas et al. [27]
Work up error/missing follow up appointments	Adult patients	Moore et al. [45]
	Adult patients	Thomas et al. [27]
Older age	Adult patients	Hallgren et al. [43]
	Adult patients	Leendertse et al. [7]
	Adult patients	Thomas et al. [27]
	Adult patients	Davies et al. [25]
Male sex	Adult patients	Hallgren et al. [43]
	Patients 60 years and older	Zhang et al. [7]
Female	Patients hospitalized in a geriatric unit	Cabre et al. [33]
Black race	Adult patients hospitalization at general medicine	Allaudeen et al. [44]
Comorbidities (including high comorbidity score)	Adult patients	Hallgren et al. [43]
	Adult patients	Leendertse et al. [7]
	Patients 60 years and older	Zhang et al. [24]
Renal disease/—insufficiency	Adult patients hospitalization at general medicine	Allaudeen et al. [44]
	Patients hospitalized in a geriatric unit	Cabre et al. [33]
	Adult patients	Leendertse et al. [7]
Congestive heart failure	Adult patients hospitalization at general medicine	Allaudeen et al. [44]
Cancer	Adult patients hospitalization at general medicine	Allaudeen et al. [44]
	Patients 65 years old or older	Hauviller et al. [28]
	Patients 80 years old or older	Alassaad et al. [34]
Iron deficiency anemia	Adult patients hospitalization at general medicine	Allaudeen et al. [44]
Presence of pulmonary disease	Patients 80 years old or older	Alassaad et al. [34]
Cognitive impairment or dementia	Patients 70 years old or older	Wimmer et al. [40]
	Adult patients	Leendertse et al. [7]
Weight loss	Adult patients hospitalization at general medicine	Allaudeen et al. [44]
Falling in the last 12 months	Adult patients	Hallgren et al. [43]
Length of stay in the hospital	Adult patients	Leendertse et al. [7]
	Patients 60 years and older	Zhang et al. [24]
Discharged to nonhome setting/Depending living situation	Patients 70 years old or older	Wimmer et al. [40]
	Adult patients	Leendertse et al. [7]
Elderly men with adult children as caregivers	Adult patients	Olson et al. [41]
Responsibility	Adult patients	Hallgren et al. [43]
Feelings of loneliness	Adult patients	Hallgren et al. [43]
Self-rated health	Adult patients	Hallgren et al. [43]
Life-Satisfaction	Adult patients	Hallgren et al. [43]

### Interventions which may reduce the medication related readmissions

Different interventions to reduce the risk of medication related admissions or readmissions are mentioned in literature. One of them is the involvement of a pharmacist as part of the medical team [46].

The effect of such an intervention is hard to evaluate due to the different ways the participation of the pharmacists is executed. Furthermore there was great difference in the way a patient is involved in his/her medication management [46–52].

Overall, studies show a possible benefit with regard to participation of a pharmacist, especially in patients with a high risk of medication related admissions, but studies show great heterogeneity [51, 53, 54].

Different studies investigated whether education could improve the medication adherence, because low and intermediate medication adherence is associated with more readmissions compared to high medication adherence [42]. Some publications show that some interventions increase the medication adherence however there is a great heterogeneity and not all methods are effective [55].

Besides education to increase the medication adherences different studies investigated whether packaging of the medication would increase the medication adherence. A meta-analysis found that packaging intervention increases medication adherence [56]. As earlier mentioned, high adherence is associated with less readmissions, this means that packaging intervention might indirectly lead to less medication readmissions.

Different education programmes and transition-of-care interventions are used in several studies; most of them show lower readmissions. However, these interventions are time consuming and the studies show great heterogeneity [55, 57, 58].

Digital assistance in the form of Clinical Decision Support Systems (CDSS), is also being investigated as intervention to improve outcome in medication related problems [59, 60]. CDSS supports the healthcare professional, pharmacist and/or physician, in optimizing medication. This system is based on a database that generate drug safety alerts for the use of medication based on different guidelines/criteria, laboratory values and patient characteristics [59, 60].

Studies show that a CDSS can support the professional in performing a medication review [59, 61]. The system is especially of additional value in recognizing absent medication when there is a clear indication and when there are contra indications or interactions for medication [59]. Another benefit of a CDSS is that the medication is monitored continuously whereas a manual medication review is performed only once or twice a year due to time pressure [59, 60]. For example, the renal function changes over time, requiring adjustment of medication which will be immediately detected by the CDSS in contrary to manual medication review.

Previous studies have shown that the use of CDDS has an additional value for the manual medication review [59, 61].

## Discussion

The aim of this literature overview was to give an overview on what currently is known about medication related hospital admissions, medication related hospital readmissions, their risk factors, and possible interventions which reduce medication related hospital readmissions. The incidence of medication related hospital admissions shows a great variety and ranges between 0.5 and 19.3% and is dependant of the definition used in the different studies [7, 10, 17–20, 22]. The incidence of medication related hospital readmissions has even a broader range, namely 0.09% up to 64.0% [27–29]. The most important identified risk factors for medication related admissions or medication related readmissions are high risk medication, polypharmacy, therapy nonadherence, older age, comorbidities, renal disease, congestive heart failure, cognitive impairment and length of stay in the hospital [7, 23–25, 27, 33, 36, 38–40, 42–44].

The most common medications associated with (potentially preventable) admissions are anticoagulants, antiplatelet drugs, vasodilators, psychotropic medications and diuretics [7, 23, 33].

However all of the results show much heterogeneity between studies. The study designs and definitions used for medication related admissions and medication related readmissions are different between the studies.

In the included studies, different interventions are investigated such as the involvement of pharmacists in medication reviews during the admissions of patients, different education programs and transition-care interventions. Some studies show less medication related readmissions, however the results are controversial. Probably due to the different methods, study populations and interventions which are investigated. For example the involvement of pharmacist in medication reviews during an admission is different in the selected articles, however overall there is a possible benefit with regard to participation of a pharmacist, especially in patients with a high risk of medication related admissions. Beside the involvement of a pharmacist in the medication reviews during the admission, other studies investigated the value of the use of CDDS. As mentioned earlier previous studies have shown that the use of CDDS has an additional value for the manual medication review [59, 61]. But the effect on the readmission rate is not known yet.

The limitation of this study is that the review was not systematic and the search was limited to the PubMED database. The aim of the study and the search were both broad, however we only performed one search. Afterwards it was possibly better to specify the aim and to convert the search for the more specific aim. With this search used for this review we found a lot of articles not related on this subject. Possibly we also missed articles on this subject because we only performed one search. The strength of this review is that this review gives an overview about a topic which is important in the daily care. Although there is a great variety in results, overall the studies show the importance to get more knowledge about this topic to prevent potential preventable unfavourable outcomes and high healthcare costs.

In the future we want to investigate the additional value of the CDSS in medication related hospital readmissions in people older than 60 years. Because there is a lack of a definition in the literature for a medication related admission and readmission, we have chosen to select unplanned admissions which are possible medication related. The Dutch guideline “Polypharmacy in the older patient” includes a trigger list that can be used to establish whether an admission is possibly medication related [62]. The trigger list is mainly based on three studies namely the HARM-, IPCI- and Quadret, and presents the most frequent medication related problems which can lead to an admission [7, 11, 63, 64].

Patients aged 60 years and older with an unplanned hospital admission will be included in the study if the unplanned hospital admission is assessed to be medication related according to the trigger list. Participants will be randomized in intervention or control group. In the control group care as usual will be continued. In the intervention group a medication check will be performed weekly using the CDSS. The generated alerts/recommendations will be sent to the general practitioner and/or home pharmacist. Follow-up will be one year.

With the assistance of the CDSS we aim at reducing the medication related readmissions from 20 to 15%.

## Conclusion

The definition for both medication related hospital admissions and readmissions varies in different studies leading to a great incidence range. Several risk factors related to medication related hospital admissions and/or readmissions have been identified: high risk medication, polypharmacy, therapy nonadherence, older age, comorbidities, renal disease, congestive heart failure, cognitive impairment and length of stay in the hospital. Known interventions that could possibly lead to a decrease in medication related hospital readmissions are spare being the involvement of a pharmacist, education programs and transition-care interventions the most mentioned ones although controversial results have been reported. More research is needed to gather more information on this topic.
